# Career Exploration of High School Students: Status Quo, Challenges, and Coping Model

**DOI:** 10.3389/fpsyg.2021.672303

**Published:** 2021-09-24

**Authors:** Huaruo Chen, Fei Liu, Ya Wen, Ling Ling, Shi Chen, Hairong Ling, Xueying Gu

**Affiliations:** ^1^School of Education Science, Nanjing Normal University, Nanjing, China; ^2^Center for Research and Reform in Education, Johns Hopkins University, Baltimore, MD, United States; ^3^School of Teacher Education, Nanjing Xiaozhuang University, Nanjing, China; ^4^Institute of Mathematics and Physics, Beijing Union University, Beijing, China

**Keywords:** career exploration, challenge, coping model, high school, China

## Introduction

Future career choices of teenagers have always been the focus of researchers (Pânişoara et al., 2013). In 2014, China proposed a new policy for the reform of the New College Entrance Examination (NCEE), which clearly pointed out that students should choose their appropriate subjects for future study and their development and put forward higher requirements for their self-interest and future career development (Wang, 2021). The new model of subjects selection provides more choices, which not only challenges schools and teachers, but also puts forward higher requirements for the ability of the students for career exploration (Jian, 2020). The selection of subjects will affect the future academic achievements, majors, and employment in universities of the students (Chen et al., 2021). Career exploration is one of the hot research topics in the field of career development in recent years (Jiang et al., 2019) and is considered as a great impetus to the adaptive development of people (Guan et al., 2018), which refers to the process that an individual explores the environment related to himself/herself and career development under the impetus of exploration motivation (mainly professional exploration in high school) (Derevensky and Coleman, 1989; Qu and Zou, 2009). Under the background of NCEE reform, it is particularly important for high school students to master and apply the ability of career exploration (Chen et al., 2021). After combing the concept, structure, measurement, and influencing factors of career exploration, this study planned to explore the challenges faced by high school students in China, put forward an opinion, and established a new coping model suitable for the career exploration of high school students, to provide help to them for discipline selection and future development.

## Literature Review

### Concept of Career Exploration

The research of career exploration originated from the theory of career development (Jordaan, 1977; Nevill, 1997; Super and Jordaan, 2007), which divided personal career development into five stages as follows: growth, exploration, establishment, maintenance, and decline (Hansen, 1993; Beale, 1998). The career exploration period (15–24 years old) is an important preparation stage (Gottfredson, 1981), and it is also an important period for high school students to know themselves and explore their majors under the NCEE (Darolia and Koedel, 2018; Liu and Helwig, 2020). Career exploration, which refers to a kind of psychological or physical activity taken by individuals to achieve career goals, including information seeking, self-cognition, and environment (Phillips, 1982; Blustein, 1992; Gross-Spector and Cinamon, 2018). Then according to this definition, scholars put forward that the process of career exploration should pay attention to three contents, as follows: information seeking, self-cognition, and environmental cognition (Blustein, 1989). Corresponding to high school students, it mainly involves several aspects, such as the exploration of University majors, the pursuit of subjects selection, self-cognition, and understanding of future University learning environments (Lau et al., 2021).

Career exploration of high school refers to a kind of physical or psychological activity that high school students carry out to achieve the optimization of future college professional learning choices, including the process of self-cognition, college professional information collection, and college learning environment cognition. In the process of exploration, the abilities of self-perception, self-reflection, self-matching, and self-regulation can be formed.

### Structure of Career Exploration

Nowadays, researchers have different views on the structure of career exploration, which mainly includes content orientation, process orientation, and general orientation (Sugalski and Greenhaus, 1986).

In terms of content orientation, career exploration is a simple behavior, including the exploration of self and surrounding environment, emphasizing the object to be explored (An and Lee, 2017). Parsons proposed that the exploration objects of individuals in career development include not only their own interests and abilities but also career-related information, which can make a reasonable match between individuals and careers (Parsons, 1909; Baker, 2009). There are also researchers in China who divide career exploration into self-exploration and environment exploration from the content (Jiwen Song and Werbel, 2007). Self-exploration refers to the development of self-information, the definition of values, interests, personality characteristics, abilities, life type tendencies, etc. (Blustein, 1989). Environmental exploration includes paying attention to and collecting information related to occupation, work, and organization (Teixeira and Dias, 2011; Xu et al., 2014).

In terms of process orientation, researchers pay close attention to the process and dynamics of career exploration, emphasize the continuous development of individuals, obtaining information, and finally determining career goals (Blustein, 1989; Ketterson and Blustein, 1997). Career exploration theory put forward that it should pay attention to the belief and process of career exploration, and focus on the initial motivation. Career exploration development theory focused on the process and reflection of career exploration and the gains after exploration. Both theories provide an important theory and foundation for this study, that is, career exploration needs to pay attention to three dimensions, including belief, process, and reflection. (Stumpf et al., 1983; Flum and Blustein, 2000).

In terms of general orientation, the meta model of career exploration is representative of integration orientation, which pointed out that there was still no clear and unified structure in the field of career exploration. Its purpose was not to present new knowledge but to organize existing information and integrate it into a systematic pattern. So a meta model of career exploration divided career exploration into two parts, namely, self and environment. There are four stages in each exploration, such as concrete experience, reflective observation, abstract conceptualization, and positive experience. The activities and tasks of individuals in exploring themselves and the environment are different (Atkinson and Murrel, 1988). To sum up, career exploration should include two subdimensions, belief and process while focusing on personal exploration and environmental exploration (Model composition 1).

### Measurement of Career Exploration

Up to now, there are more and more empirical studies on career exploration, but no measurement tools of various types. The main research types can be divided into three types, namely, career exploration survey (CES), career development inventory (CDI), and self-developed measures.

In terms of CESs, with the maturity of the theoretical research on career exploration, researchers have developed several scales to test the level of career exploration. Stumpf et al. (1983) developed the 59-item CES to capture three major categories of exploration, including the exploration process, reactions to exploration, and beliefs about exploration (Stumpf et al., 1983). Since its establishment, the CES has become the leading means of career exploration. On the one hand, more and more empirical studies have completely adopted all the topics of CES and obtained different experimental results (Nauta, 2007; Praskova et al., 2015; Lent et al., 2017). On the other hand, many studies adjust and delete items in the subdimensions of self-exploration and environmental exploration to suit different types of subjects (Werbel, 2000; Zikic and Klehe, 2006). However, the long process of CES leads to its limitations in empirical research. Although more and more researchers focus on one of the subdimensions, it cannot solve the overall understanding of career exploration (Jiang et al., 2019). Therefore, the recent research began to seek a shorter experimental way of career exploration.

In terms of CDI, it is an American instrument designed to measure the vocational maturity of adolescents (Super, 2007; Hansen, 2018). Resource search, one of its subscales, is often used to measure career exploration. Since the scale has many topics and different measurement contents, later researchers developed a shorter scale, which has also been used in empirical studies from many countries, including Australia (Patton et al., 2004; Rogers et al., 2018), Swiss (Hirschi, 2011), Italy (Chiesa et al., 2016), China (Chen et al., 2021), and so on. Compared with CES, CDI emphasizes the exploration or thinking of external resources rather than individuals in the process of career exploration (Jiang et al., 2019). Another problem worthy of pointing out is that the validity of this subscale of CDI is questionable because most projects involve how the information sources are provided, or belief in work or occupation, so there is no clear assessment of the degree of active participation of individuals in career exploration. In view of these limitations, researchers should be cautious when using the career exploration subscale of CDI as a research tool in future work.

In addition to the above two main measurements, researchers have developed some self-developed measures to evaluate career exploration. Most of these CES subscales have something in common, mainly including self-exploration and environmental exploration, which are particularly related to behaviors in the process of career exploration but pay little attention to attitudes and beliefs. The existing self-developed measures mainly include 6-item scales (Yuen et al., 2010; Rojewski et al., 2014), 13-item scales (Tracey et al., 2006), and 24-item scales (Vignoli et al., 2005). However, unlike CES and CDI, these new measures have not been widely adopted. It is worth noting that the existing research on career exploration of high school students mainly focuses on the measurement of exploration behavior and belief (Phillips and Blustein, 1994; Brown et al., 1999), and has not developed a scale or interview more suitable for high school students. To sum up, career exploration should include reflection (Model composition 2).

### Influencing Factors of Career Exploration

The research on the influencing factors of career exploration mainly focuses on family and individual factors (Ingrid, 2004), and this study mainly reviews the research in this field from these two aspects. However, in the background of the NCEE, the importance of peer groups and schools to individual career exploration, especially for high school students, cannot be ignored, which is a very important part of future research and practice (Lazarides et al., 2015).

In the past, the research on family factors mainly includes two types: construct variables and process variables. On the one hand, there are some evidence which showed that family economic status, working status of parents, and education level can affect the career exploration of teenagers. For example, family economic status was confirmed as a powerful predictor of career exploration and choice, which pointed out middle-class parents will consciously participate in the development of children and provide them with an activity place for interest and ability development, whereas working-class parents tend to regard the development of children as a natural process (Phillips et al., 2002). The work experience, employment status, and working conditions of parents have a direct or indirect influence on the career exploration and development of teenagers (Corey and Chen, 2019; Wang et al., 2019). On the other hand, it involves parent–child attachment, parental support, and parenting style (Lindstrom et al., 2007; Gagnon et al., 2019). For example, parental behaviors predicted the change in career exploration of German ninth graders over the observed period. Moreover, frequent talks with peers about career-related issues were significantly associated with the intensity of information-seeking behaviors and, at the same time, predicted intensification of occupational exploration during the following 6-month period (Kracke, 2002). These two aspects influence each other and play an irreplaceable role in the career development of teenagers.

Previous studies have shown that individual factors affecting career exploration mainly include gender, identity, personality, individual development initiative, career self-efficacy, and so on (Wang et al., 2019; Chen et al., 2020a,b). For example, compared with boys, girls reported more negative certainty and expectation of results. Moreover, girls show less satisfaction with the information they get and experience more pressure to explore and make decisions (Lim and Lee, 2021). In addition to the Big Five factors, optimism and self-esteem are also the personality factors concerned. Nauta (2007) explored the relationship between the five personalities of college students and career exploration, and found that there was no significant correlation between personality and environmental exploration, whereas openness was positively correlated with self-exploration, and extroversion was negatively correlated with self-exploration (Nauta, 2007). Mota et al. (2012) pointed out that there is a correlation between career exploration, career decision-making difficulties, and career indecision level of 9th-grade Portuguese students. This intervention can effectively enhance the understanding of teenagers of career roles and help them make active decisions (Mota et al., 2012). However, there are different conclusions. Reed et al. (2004) found that responsibility and extroversion are positively correlated with environmental exploration, and openness is positively correlated with self-exploration (Reed et al., 2004). To sum up, career exploration should include personal characteristics (such as interests and personality) and environmental factors (such as University and professional information) (Model composition 3).

## Methodology

**Figure 1:**
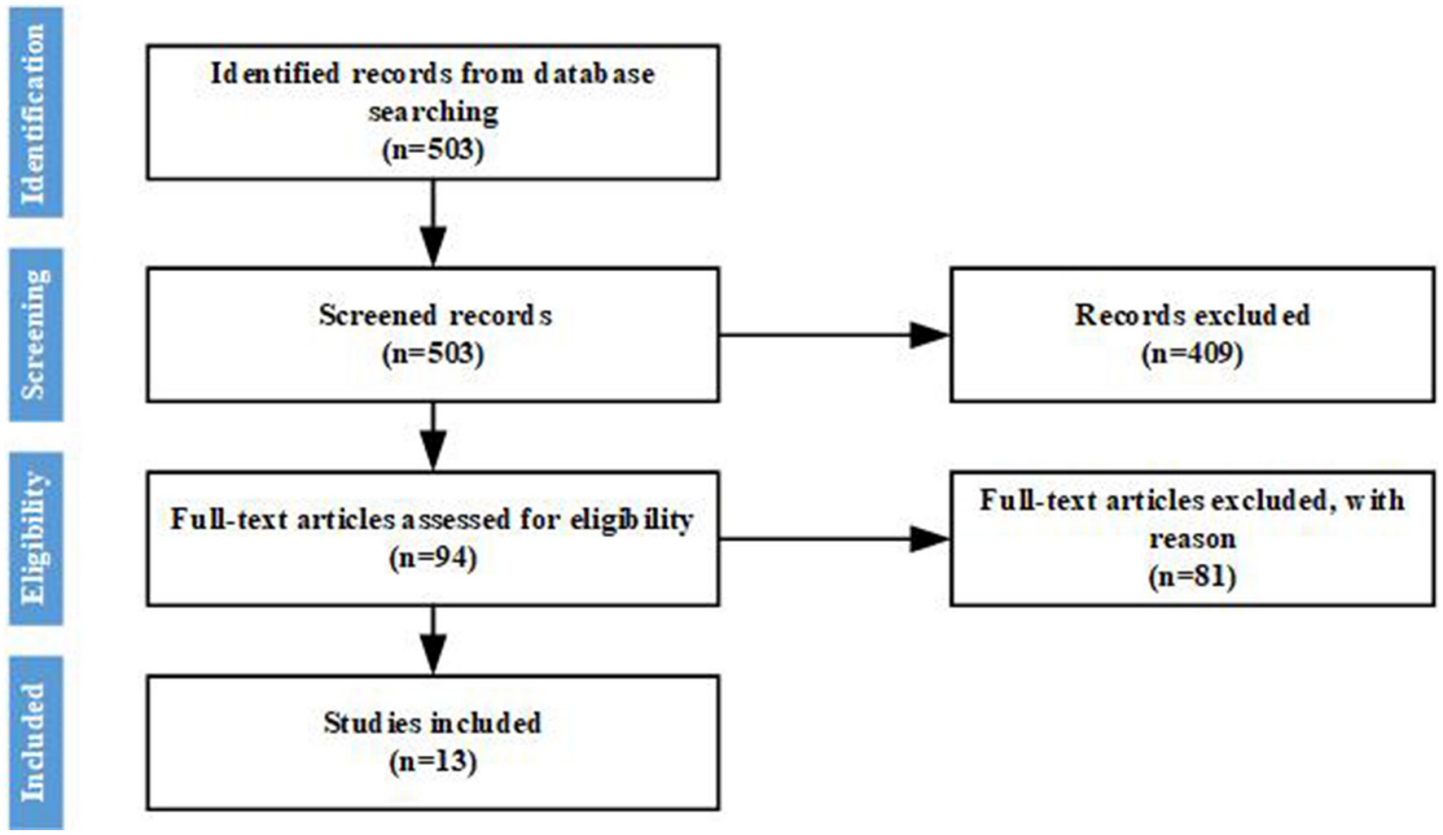
Flow chart of the study selection process.

In order to effectively collect literature on career exploration in high schools, this study was carried out by following the reporting checklist of the Preferred Reporting Items for Systematic Reviews and Meta-analysis (PRISMA) guidelines (Moher et al., 2014; Chen et al., 2020a). The literature search, screening, and clustering methods employed in the systematic review are summarized in Figure 1 and described in more detail below.

### Literature Search

The purpose of the systematic review is to discover the development status and challenges of career exploration in high schools. Three groups of search terms are defined, which are as follows: “career exploration,” “new college entrance examination,” and “ subjects selection.” The list of search terms includes words related to all learning theories known by the author. Search is limited to articles written in English and Chinese from Chinese authors and published from January 2000 to July 2021. The data come from Web of Science and CNKI. The initial literature search was conducted from January 2010 to March 2020 and then supplemented in July 2021. The initial search produced a total of 503 papers (235 from Web Of Science and 268 from CNKI).

### Screening of Titles and Abstracts

The titles and abstracts of the articles identified by the literature search were screened by hand using the following inclusion and exclusion criteria: (1) research topics must be career exploration, college entrance examination, personal exploration, or University major selection; (2) the research object must be Chinese high school students; (3) papers must be published in journal articles, conference documents or dissertations, etc.; (4) the article must be in English or Chinese. Screening of titles and abstracts reduced the set of relevant articles to 94.

### Screening of Full Articles

Two authors were assigned to each of the learning theories. Both authors read all of the articles in their category and excluded any article that was deemed not relevant to career exploration. Articles that included high school students were not excluded if they also included younger or older participants. Both theoretical and empirical studies were included. Screening reduced the set of relevant articles to 13.

## The Challenges in Career Exploration of High School

After analyzing the literature review and the literature collected about career exploration in this study, this study summarizes it into three challenges, which are described as follows:

### Not Enough Attention to the Career Exploration of High School

Under the premise of realizing self-cognition, high school students can stimulate their inner needs and become interested in themselves, thus forming the inner motive force for career exploration (Creed et al., 2007). The career planning education of high school includes five basic contents, such as self-cognition, academic mastery, major exploration, career planning, and life planning (Gu et al., 2020). Through the six steps of “self-awareness, world awareness, the initial establishment of career goals, decision-making, action and implementation, evaluation and adjustment,” students can not only cultivate the ability of career planning with the core of choice ability but also cultivate core skills and excellent character to adapt to future career changes (Rogers et al., 2008). These are only important parts of the career exploration period, which also shows that career exploration should be an important part of career education in high school. The emphasis should be placed on the guidance of self-cognition (Chen et al., 2020a). However, this study found the following: (1) facing the research of career exploration, more people pay attention to how graduates make the best choices, rather than looking for the reasons that make career choices difficult (Chan, 2018); (2) facing the development of high school students, more and more people choose to pay attention to academic achievements (Otte and Sharpe, 1979; Masud et al., 2019), family support (Pires et al., 2017), and school conditions (Lombardi et al., 2019). To sum up, although the attention to the career exploration of high school has risen in recent years, it is still difficult to maintain high attention and in-depth theoretical research, and many studies are borrowed from other research objects without considering the special situation of high school students. To sum up, the main development of career exploration depends on the family and school support (Model composition 4).

### No Practical Action to Explore University Majors

Taking NCEE in China as an example, the proposal of a new elective mode is particularly crucial for the exploration of University majors (Liu, 2014; Zhang, 2019). However, there is little research in this field. Currently, it mainly involves professional classification and research trips. In the professional classification, Xie (2016) points out that there are currently about 500 majors in colleges and universities in China, which are divided into 18 majors according to their similarities (Xie, 2016). At the same time, through understanding the world map of the 18 academic groups, GPS positioning of academic groups, enrollment promotion meeting, professional GPS positioning, and other links, high school students can explore University majors. In terms of the research trip, Yang pointed out that as an effective complement to school education, research trip has strengthened the connection between schools and society, curricula and the 'lifeworld of students, organically combining tourism, study, and research, helping students understand University majors and promoting their all-round development (Yang, 2018).

To sum up, the premise of carrying out research and study travel activities is the need to correctly understand the basic nature and practical role of its professional exploration in universities (Huang et al., 2016). Designing rich tourism courses and improving the system and mechanism of cooperative organization and management is the cornerstone of carrying out activities. However, it is a pity that although some studies have pointed out the importance of this measure, no pertinent suggestions or practical results have been made. To sum up, the career exploration of high school mainly focuses on the professional exploration of University, and the school should provide corresponding courses as an auxiliary (Model composition 5).

### Lack of Operating Mechanism of Environmental Cognition

Environmental cognition, as a very important part of career exploration, is the psychological basis for individuals to adapt to and act on the environment (Henry and Dietz, 2012). How to understand the environment? How to form an impression of the environment in your mind? How do you affect how individuals work with the environment? These issues have evolved into studies in the field of environmental psychology, such as city and architectural images, cognitive maps, and road exploration (Silvianingsih et al., 2019).

Environmental cognition of returning to high school mainly includes cognition of high school learning environment, experience, and cognition of future professional learning environment of the University. However, little is known about the research in this field. Only Song in 2011 put forward the correct understanding of the environmental education in high school geography teaching research to effectively promote the formation of environmental protection values and sustainable development of students (Song, 2011). Therefore, when it comes to environmental cognition, career exploration should learn more from the relatively mature environmental psychology at the level of smashing psychology and carry out deeper exploration. To sum up, career exploration in high school needs to pay attention to personal characteristics such as personal professional values and practical experience (Model composition 6).

## Discussion

### Research Findings

#### Literature Characteristics

Based on the analysis of the characteristics of the last 13 papers, this study found the following: first, the documents lack a strong theoretical basis, and most of the papers do not adopt the characteristics of high school students but directly adopt the theory of adult career exploration, which leads to the inability to teach students in accordance with their aptitude; secondly, among these 13 papers, six English papers are empirical research and journal articles, and seven Chinese papers are theoretical research and mainly dissertation. This reflects that the career exploration research in high school is paid attention to and the research is scattered, which is most likely caused by the lack of mature career exploration guidance in high school; and finally, all the 13 documents were published after the reform of the new college entrance examination of China in 2014, which shows that career exploration started in China not long ago, and so it is extremely urgent to provide a reference coping model.

#### Literature Content

Based on the analysis of literature and practice, this study found the following: (1) there is little research on career exploration in high schools, and a unified concept definition has not been formed yet; (2) all empirical studies are focused on some practical operation, lacking systematic and complete career. Therefore, this study attempts to establish a coping model of career exploration in high schools to explain the implementation of career exploration in high schools, which has important theoretical and practical significance.

### Coping Model of Career Exploration

**Figure 2:**
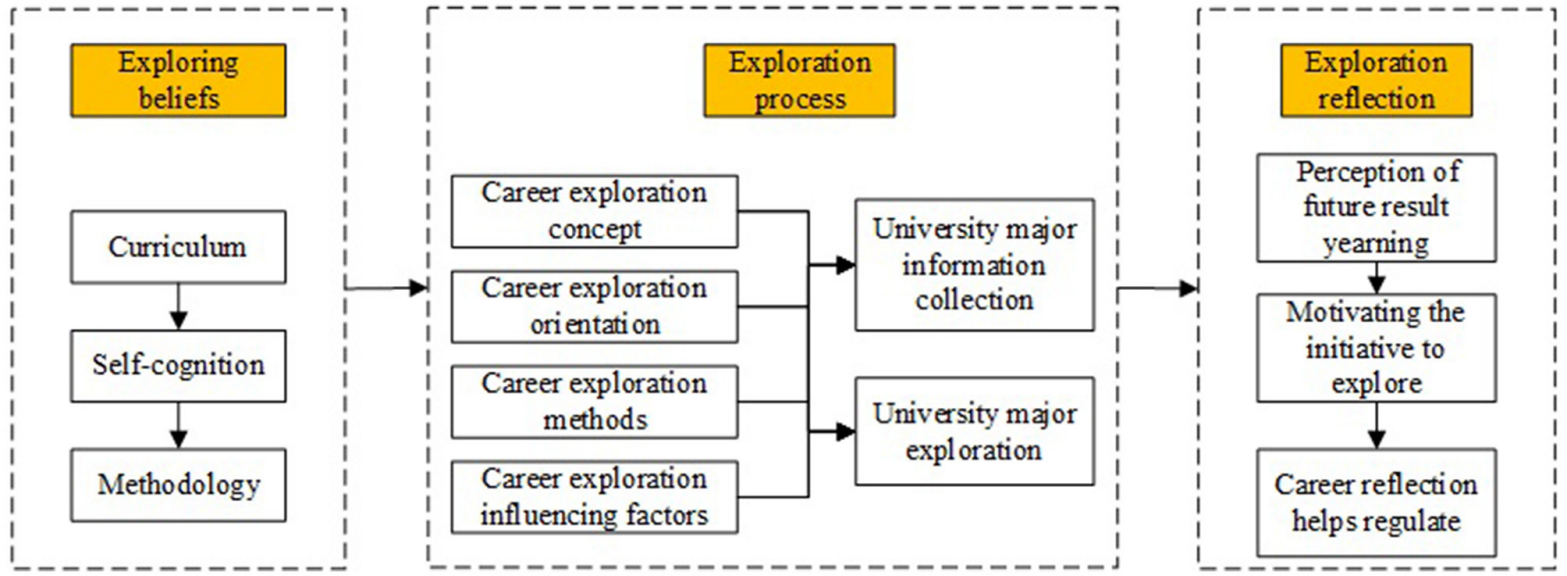
Coping model of career exploration for high school students.

On the practical level of career exploration, the Chinese researchers are basically in the process of focusing on school activities, and rarely discuss the actual needs of high school students under the NCEE (Chen et al., 2021). Even though some studies suggested the necessity of career exploration of high school students (Xie, 2016; Chen et al., 2020a), there were no effective measures to form the concept of exploration for high school students who were in the process of experience, even after tracking the experience. Therefore, the greatest practical value in this study lied in trying to establish the coping model of the career exploration of high school students based on the actual needs of high school students in China. As shown in Figure 2, the final coping model is formed by synthesizing the conclusions of the 6-model composition mentioned in the full text, which is helpful for high school students to have a reference sequence and theoretical basis in the process of career exploration, thus realizing effective topic selection and career exploration under NCEE in China.

Firstly, establish the career exploration beliefs. Due to the characteristics of high school students, taking schools as the main body and teaching through courses is considered as one of the more effective measures to solve the career exploration and future career preparation of Chinese high school students (Gu et al., 2020; Chen et al., 2021). Therefore, this study believes that the knowledge and information of career exploration under NCEE can be taught to high school students through career courses so as to use career-related means to clarify self-awareness, understand their needs and interests, and form a basic methodology. It should be noted that, in the practice of this study, it is found that all the teaching can be completed with the help of assessment and other technologies so as to ensure that students can form a methodology of self-cognition and career exploration.

Secondly, experience the career exploration process. Through the establishment and understanding of the connotation, structures, methods, and influencing factors of career exploration, the career exploration process is aimed at the needs of high school students, especially at the important stage of entering University. It can develop the ability to self-collecting University major information, combine the part of self-cognition, analyze and adapt to our University majors, and purposefully understand the future courses, learning, occupation.

Finally, make the career exploration reflection. Through the exploration of the above two steps, high school students can build up self-confidence, yearn for and perceive a better future, and thus further stimulate the motivation of career exploration, and through each stage of career reflection, make appropriate adjustments to the exploration process to achieve more suitable development.

## Conclusion

Career exploration in high schools is a rising star in China, especially under the impetus of the new college entrance examination reform policy. The new college entrance examination requires students to choose subjects in advance, which promotes students to explore their careers in time so that they can make better decisions and lay a good foundation for future career development. Therefore, after sorting out the concept, structure, measurement, and influencing factors of career exploration, this study uses the method of systematic review to screen articles for analysis and points out the great challenges it brings to career exploration. In addition, this study takes the reform of NCEE in China as an example and pays more attention to the reaction, in addition to the process and belief that should be paid attention to in the reflection of career exploration. From these three dimensions, it puts forward relevant coping models and guidelines for supporting the development of high school career exploration, which provided a theoretical model and coping path for the practice of high school career exploration. Although there is a serious lack of research on career exploration in high school in the existing literature, with the promulgation of various policies, more and more researchers realize the importance of career exploration. As this study only puts forward an opinion, it does not give guidance or form an action guide from the practical level, which is the focus of future research.

## Author Contributions

All participated in the study design. HC, FL, YW, LL, and XG wrote the first draft. SC and HL modified the manuscript.

## Funding

This study was funded by Project of Scientific Research Innovation plan for Postgraduates in Jiangsu Province, Grant Number KYCX20_1145, Jiangsu Province Basic Education Prospective Teaching Reform Experiment Project, and Jiangsu Province University's Advantageous Discipline Construction Project, Grant Number PAPD.

## Conflict of Interest

The authors declare that the research was conducted in the absence of any commercial or financial relationships that could be construed as a potential conflict of interest.

## Publisher's Note

All claims expressed in this article are solely those of the authors and do not necessarily represent those of their affiliated organizations, or those of the publisher, the editors and the reviewers. Any product that may be evaluated in this article, or claim that may be made by its manufacturer, is not guaranteed or endorsed by the publisher.
